# Ψ-RA: a parallel sparse index for genomic read alignment

**DOI:** 10.1186/1471-2164-12-S2-S7

**Published:** 2011-07-27

**Authors:** M Oğuzhan Külekci, Wing-Kai Hon, Rahul Shah, Jeffrey Scott Vitter, Bojian Xu

**Affiliations:** 1 National Research Institute of Electronics & Cryptology, 41470, Gebze, Kocaeli, Turkey; 2 Department of Computer Science, National Tsing Hua University, Hsinchu, Taiwan 30013, R.O.C; 3 Department of Computer Science, Louisiana State University, Baton Rouge, LA 70810, U.S.A; 4 Department of Electrical Engineering and Computer Science, The University of Kansas, Lawrence, KS 66045, U.S.A

## Abstract

**Background:**

Genomic read alignment involves mapping (exactly or approximately) short reads from a particular individual onto a pre-sequenced reference genome of the same species. Because all individuals of the same species share the majority of their genomes, short reads alignment provides an alternative and much more efficient way to sequence the genome of a particular individual than does direct sequencing. Among many strategies proposed for this alignment process, indexing the reference genome and short read searching over the index is a dominant technique. Our goal is to design a space-efficient indexing structure with fast searching capability to catch the massive short reads produced by the next generation high-throughput DNA sequencing technology.

**Results:**

We concentrate on indexing DNA sequences via sparse suffix arrays (SSAs) and propose a new short read aligner named Ψ-RA (PSI-RA: parallel sparse index read aligner). The motivation in using SSAs is the ability to trade memory against time. It is possible to fine tune the space consumption of the index based on the available memory of the machine and the minimum length of the arriving pattern queries. Although SSAs have been studied before for exact matching of short reads, an elegant way of approximate matching capability was missing. We provide this by defining the rightmost mismatch criteria that prioritize the errors towards the end of the reads, where errors are more probable. Ψ-RA supports any number of mismatches in aligning reads. We give comparisons with some of the well-known short read aligners, and show that indexing a genome with SSA is a good alternative to the Burrows-Wheeler transform or seed-based solutions.

**Conclusions:**

Ψ-RA is expected to serve as a valuable tool in the alignment of short reads generated by the next generation high-throughput sequencing technology. Ψ-RA is very fast in exact matching and also supports rightmost approximate matching. The SSA structure that Ψ-RA is built on naturally incorporates the modern multicore architecture and thus further speed-up can be gained. All the information, including the source code of Ψ-RA, can be downloaded at: http://www.busillis.com/o_kulekci/PSIRA.zip.

## Background

The last decade has witnessed a rapid development in DNA sequencing by the introduction of next generation high-throughput DNA sequencing [1] technologies. The equipment based on that new technology produces billions of reads in a single day per machine [2]. The most important two problems regarding the DNA sequencing are alignment and assembly [3]. If the target specie has not been sequenced before, a *de novo* DNA assembly [4], which requires concatenation of the reads in an optimum way, has to be performed. Otherwise, reads are mapped against a reference genome that is the result of a previous sequencing effort of the same specie. With the advent of the next-generation sequencing, various short read aligners such as Bowtie [5], PerM [6], SOAP [7], mrs-Fast [8], and many others have been proposed in the last three years. In a recent study, Li and Homer [9] surveyed short read aligners in general.

Many strategies have been applied to perform the alignment process fast and accurately. While some of the aligners index the reference genome, some others rely on hash tables based on q-grams or spaced seeds to perform a quick scan. Although hash-based solutions are more flexible in detecting approximate matches, indexing solutions are faster. The dominant solution in genome indexing is the Burrows-Wheeler transform (BWT) [10] of the reference sequence (e.g., [5,11]), in which the reads are searched with the backwards search algorithm introduced in FM-index of Ferragina and Manzini [12].

We concentrate on indexing the genome via sparse suffix arrays (SSAs). Lexicographic ordering of all the suffixes of the text forms the suffix array [13], which is a well-studied data structure initially proposed to lower the space requirement of the suffix tree. Although suffix arrays are much more space-preserving than suffix trees, they still require large memory space for indexing massive data such as the whole genome of a human. The space consumption of an ordinary suffix array for a given text of *n* characters is *O*(*n* log *n*)*.* The occurrences of a pattern of length *m* characters can be found in *O*(*m* log *n*) time by searching the pattern on the suffix array by binary search procedure. This bound can further be improved to *O*(*m* + log *n*) time by using auxiliary data structures [13].

It was long believed that compressing a suffix array is not feasible since it is mainly a permutation of the numbers from 1 to *n.* Grossi and Vitter [14] refuted that belief and showed that suffix arrays are compressible. Following that work, compressed data structures in text indexing gained greater focus within the community [15,16]. These efforts resulted in showing that an index for a text can occupy space proportional to the compressed size of the text itself. However, if it is not possible to significantly compress the input data, as is the case for DNA sequences, these methods do not provide a considerable advantage.

Recently, Chien *et al.*[*17*] proposed *sparsíficatíon* of the suffix array as an alternative method to compress it. The key idea is that instead of sorting all the suffixes beginning from each position 1 to *n* of the text, a sorted list is created of the suffixes that begin at positions *p*, such that *p* mod *D* = 0, for all 1 ≤ *p* ≤ *n*, where *D* denotes the sparsification factor. As a result, we have a list *of n*/*D* numbers in the sparse suffix array rather than *n* numbers in the original suffix array. We interpret the text with a new alphabet by combining each *D* contiguous original characters into a meta-character. The length of the text is now *n/D* meta-characters. Thus, the space consumption decreases to *O*((*n/D*) log(*n/D*))*.*

The main two drawbacks of the sparse suffix arrays are i) the necessity to run the search procedure *D* times, and ii) a complicated search procedure when the queried pattern length is less than *D.* The necessity to run the search procedure *D* times comes from the fact that the queried pattern may begin at some position s, where the value *s* mod *D* can be any one of *D* different values. Thus, *D* appropriate alignment positions should be checked one by one (see the next section for a more detailed explanation).

When the pattern length is shorter than the sparsification factor *D*, then the meta-characters, which are *D* ordinary characters in length and may include the queried pattern, must be investigated first. Specified meta-characters should then be located on the text to finalize the actual search (see [17] for detailed analysis of the case), which is not so elegant.

On the other hand, we have two advantages to overcome these drawbacks, in particular for the short read alignment problem. First, today's multicore processor architecture enables parallelism more than ever. We can benefit from multicore architectures to decrease the overhead in repeating the search procedure *D* times. Second, next-generation sequencing machines have a lower bound on the length of the reads. Short reads are in range from 25 to 100 base pairs (bp), and it is foreseen that lengths will be longer in a couple of years. Thus, if we choose the sparsification factor *D* less than the minimum possible length of the input reads, we do not need to deal with short pattern case anymore.

Recently, Khan *et al.*[18] proposed using sparse suffix arrays for finding maximal matches in large sequence data along with an application on short read alignment. Their study considers only exact matching of the reads. Although it is argued in some studies [19] that exact matching would be enough for short read alignment, an approximate alignment of a queried pattern would help on reducing the total number of required reads (*coverage*) for whole sequencing. That is because the percentage of aligned reads will be lower if we neglect approximate matches.

Based on the fact that errors are more probable towards the end of the reads, we extend exact matching with sparse suffix arrays to include any number of mismatches by defining the rightmost mismatch criteria. We prioritize the errors on the right-hand side of the reads, and detect the *k*-mismatch alignments sorted according to their rightmost occurrences in an elegant way. When equipped with mismatch detection capability, sparse suffix arrays serve as a good alternative to BWT-type genome indexing, as shown by experimentally comparing the proposed sparse suffix arrays against BWT-type genome indexing.

## Methods

### Aligning with exact matches

Let *G* = *g*_0_*g*_1_*g*_2_ … *g_n_*_–1_$ be an *n*-base-long DNA sequence, where each base *g_i_* is from alphabet Σ = {A, C, G, T} and the end of the sequence is marked with a special character $ that is lexicographically smaller than all the characters in Σ. If we denote the *n* suffixes of such a given sequence by *s*_0_ to *s_n_*_–1_, then the *i^th^* suffix *s_i_* will correspond to *s_i_* = *g_i_g_i_*_+1_…*g_n_*_–1_$*.* The lexicographical sorting of those suffixes creates the suffix array *A* = *a*_0_*a*_1_…*a_n_*_–1_ such that *a_i_* is the beginning position of the *i^th^* smallest suffix of *G*, for 0 ≤ *i* <*n.*

**Figure 1:**
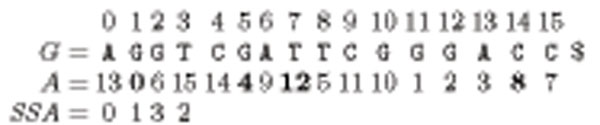
An example DNA sequence *G*, suffix array of *G*, and sparse suffix array of *G* by assuming sparsification factor *D* = 4.

The sparse suffix array (SSA) of *G* includes only those *a_i_* numbers in suffix array *A* that are multiples of a specific number *D*; that is *a_i_* mod *D* = 0. In other words, rather than sorting all the suffixes of *G*, we sort only those suffixes *s_i_*, where *i* mod *D* = 0 and keep their starting positions divided by *D* in SSA. We refer to *D* as the sparsification factor. Figure 1 depicts the suffix array and the sparse suffix array of an example sequence AGGTCGATTCGGGACC. The first element of the suffix array is *a*_0_ = 13 as *s*_13_ = ACG$ is the smallest among all suffixes of *G.* When we want to sparsify that suffix array with a factor of *D* = 4, we take only those points from *A* where *a_i_* mod 4 = 0. We preserve their order of appearance in the original suffix array. Thus, the sparse suffix array of the given *G* is *SSA* = {0,1,3,2}.

The space required by a sparse suffix array of a sequence of length *n* characters is *O*((*n*/*D*) log(*n*/*D*)). When *D* = 1, this complexity converges to that of the ordinary suffix array. The decrease in the size of the index comes with a cost in the search complexity.

The time complexity to locate all possible occurrences of queried pattern *P* on a sequence of length *n* using the ordinary suffix array is *O*(|P| · log *n*). When we use the sparse suffix array, however, the same procedure only lets us detect those occurrences starting at positions that are multiples of *D* because SSA includes the sorted list of the suffixes beginning only on those locations. We also need to consider the cases where *P* starts at positions that are not divisible by *D.*

Given a pattern *P* = *p*_0_*p*_1_*…p_m–_*_1_, where *D* ≤ *m* ≤ *n*, let  denote the initial *i* characters and  show the rest. If we search  on *G* via the SSA for each 0 ≤ *i* <*D*, and then verify whether *G* has the corresponding  preceding the positions where  appears, we can locate all possible occurrences. Algorithm 1 describes this procedure.

The time complexity of exact match via SSA is *O*(*m* log(*n*/*D*) + (*m* – 1) log(*n*/*D*) + *o*_1_ · 1 + (*m* – 2) log(*n*/*D*) + *o*_2_ · 2 + … + (*m* – *D* – 1) log(*n*/*D*) + *o_D_*_–1_ · (*D –* 1)), where *o_i_* is the number of occurrences of  on *G.* The terms beginning with *o_i_* in the summation correspond to the verification cost. Thus, total time may be approximated as *O*(*Dm* log(*n*/*D*)+ *Verification*)*.* Note that increasing sparsification factor *D* decreases the space complexity while increasing the time complexity. This gives us the flexibility to tune *D* according to the available processors and memory.

### Aligning with mismatches

Reads may contain some errors. Although today's sequencing machines produce quality values that represent the confidence of the individual bases in each read, these values vary greatly and require a fine-tuning step to be integrated into the alignment process. Using these quality values also decreases the speed of the alignment [9]. In general, the errors are more probable towards the end of the reads; hence, we define the rightmost criteria to prioritize these positions and find the *k*-mismatch approximate matchings of a given pattern.

**Figure 2:**
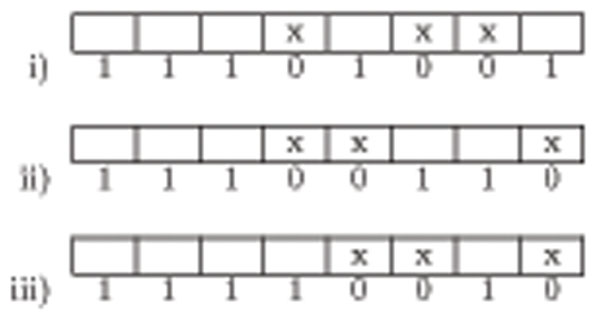
Positions marked with x indicate mismatch. When sorted according to the rightmost mismatch criteria, iii) has the first priority, followed by i) and ii) respectively.

Let a given pattern *P* = *p*_0_*p*_1_*…p_m_*_–1_ be aligned with the text segment *g_i_g_i_*_+1_…*g_i_*_+_*_m_*_–1_, for some *i*, 0 ≤ *i* ≤ (*n* – *m*), and *B* = *b*_0_*b*_1_*…b_m_*_–1_ be a binary number of *m* bits such that, for all *j*, 0 ≤ *j* <*m*, if *p_j_* = *g_i_*_+_*_j_*, then *b_j_* = 1, else *b_j_* = 0. The total number of 0-bits in *B* is the total number of mismatches between *P* and *G*[*i*,*…*,*i* + *m –* 1]. Among the possible -mismatch alignments of *P* onto text *G*, the ones having the highest *B* numbers are defined as the rightmost *k*-mismatch alignments of *P.* Figure 2 depicts this definition.

When we search a pattern by using the suffix array, the process returns a range *R* = [*sp*, *ep*] that means *P* starts at text positions *g_A_*_[_*_sp_*_]_, *g_A_*_[_*_sp_*_+1]_, *…*, *g_A_*[*ep*]. If there are no occurrences of *P* on *G*, then *ep* is one less than *sp* (see suffix range search procedure in [16] for more detail). In that case, either the *G*[*A*[*sp*],*…*, *A*[*sp*] + *m –* 1] or *G*[*A*[*ep*],*…*, *A*[*ep*] + *m* – 1] have the longest match with *P.* That is because the suffix array is a sorted list of the suffixes, and since we are running a binary search on the suffix array, the final position is the closest point to the pattern, if not itself exactly. We compare *P*[0,…, *m* – 1] against both and find for each the number of matching nucleotides from the left side until we encounter the first mismatch. We denote the maximum of these two numbers as *L*, meaning the length of the longest matching prefix of *P.* That indicates *P*[0,…, *j*] does not occur in the text for all *j* ≥ *L*, while the prefixes *P*[0,…, *i*] exist for all *i* <*L.*

For example, let's assume sample pattern AGGTCGATTCGGGACC does not occur in the reference genome, and we detect that the length of the longest matching prefix is *L* = 10, which indicates the prefix AGGTCGATTC exists in the text, but the longer prefixes AGGTCGATTCG, AGGTCGATTCGG, …, AGGTCGATTCGGGACC do not. If we attempt to alter the sample pattern's last base C to any of the A, G, or T, none of them will report a match since we know the nonexistence of its prefix AGGTCGATTCGGGAC. Similarly, changing the previous base C also does not make sense as AGGTCGATTCGGGA is absent in the reference. The first base that has a chance to match when altered is *p*_10_ = G preceded by the prefix AGGTCGATTC that occurs in the text. Thus, when looking for the 1-mismatch alignment of the sample read, we don't need to spend time to check possible alternatives of the last five bases.

Remembering the fact that *k*-mismatch alignments are 1-mismatch apart from the (*k* – 1)-mismatch alignments, we can generate the 2-mismatch patterns from the 1-mismatch cases, and keep going up to *k*-mismatch alignments. This procedure ensures the rightmost mismatch criteria.

Algorithm 2 depicts the *k*-mismatch idea in pseudocode. We keep possible alterations of original *P* in a list (*α*-list and *β*-list in lines 7 and 16) consisting of three attributes. The first one is the altered pattern. The second attribute is the list of the altered positions that are used in sorting the alterations according to rightmost mismatch criteria. Note that this inner list contains *k* numbers for *k*-mismatch alterations because a *k*-mismatch occurrence requires changing *k* positions. The third one is the offset indicating the length of the *head* of *P* as it is required in the sparse suffix search process. After initializing the *α*-list for the 1-mismatch case within the first for loop, we generate the possible *k*-mismatch alterations of *P* sorted according to the rightmost mismatch criteria by the second for loop, and then search the exact occurrences of those altered patterns on *G* by using the proposed *SSA* index.

### Aligning in parallel

Indexing via SSAs gives users the opportunity to decrease the size of the index according to the preferred sparsification factor *D.* However, the search process should consider the possible *D* offset alignments of the queried pattern as described earlier in the proposed method, which causes an increase in the time complexity with a factor of *D.* The procedure to check each offset-*i*, for 0 ≤ *i* <*D*, is independent from each other and, hence, can be executed concurrently in a multiprocessor environment. Assuming a system with *p* processors, if the sparsification factor is chosen to be *D* ≤ *p* and each offset-i search is run separately in the dedicated processor, then the increase in the time complexity can be reduced significantly in practice.

It is noteworthy that data level parallelism is always possible by distributing the number of entities among available processors. Such a multi-thread execution is nearly supported by all aligners. On the other side, algorithmic level parallelism is not that simple to achieve. By algorithmic parallelism we mean the case that even a single query is to be executed in parallel. The search mechanism of SSAs serves as a good basis for algorithmic level parallelism due to its structure partitioning the search process into *D* number of offset-*i* investigations. Thus, it is possible to parallelize even a single search query by dedicating an individual processor to each offset-*i* search. However, when the number of patterns is large, as is the case in a DNA alignment problem, it might be more advantageous to prefer data-level parallelism and simply partition the patterns among processors. In a scenario where a server is answering pattern matching queries arriving sequentially, algorithmic level parallelism will be more appropriate.

### Implementation

We have implemented Ψ-RA in C++ language based on the proposed method and used GNU compiler g++ with all optimization flags turned on. The index of a reference genome in Ψ-RA has two parts. One is the sequence itself coded in 2-bit format; the other is the sparse suffix array of the sequence with a given spar-sification factor. We use Yuta Mori's implementation (http://sites.google.com/site/yuta256/sais) of the SAIS [20] algorithm for constructing the sparse suffix array. Initially we run SAIS on the 2-bit coded sequence that actually produces sparse suffix array with *D* = 4 as each byte is composed of 4 bases. According to the input *D* parameter, we extract the required SSA from the one with *D* = 4. The input *D* parameter should be a multiple of 4 in the current implementation.

As an example, the size of the whole human genome, which has approximately three billion bases, is about 700MB in 2-bit format. If *D* = 8, SSA requires storing one-eighth of the three billion positions, which is roughly 325K numbers. Since we store each number as a 32-bit integer, SSA for *D* = 8 needs approximately 1.5GB of memory. Thus, the total size of the index becomes 2.2GB including the original sequence.

The backbone of Ψ-RA is the binary search over the SSA. We use a trick to speed up the search process on the suffix array. We *partition* the suffix array according to an initial *K* = 8 bases. That is, we store the range of each 8-gram on the SSA as a separate table that has 4^8^ = 64K rows, where each row includes the starting and ending positions of the corresponding item on the SSA. When we search the pattern ACGTTGCAGGTCA on the SSA, as an example, we first fetch the range of the first eight bases ACGTTGCA from the table and then run the ordinary binary search on that interval instead of the whole array. This trick decreases the time complexity *O*(log *n*) of binary search to *O*(log(*n*/4*^K^*)) along with the additional cost of storing the table of size *O*(4*^K^*)*.*

Ψ-RA supports *k*-mismatch search for any *k* value, but having larger *k* values requires longer times, as in other aligners. The difference is Ψ-RA returns the queried number of alignments sorted in rightmost mismatch criteria and guarantees to find them, as opposed to some aligners sacrificing accuracy for speed. The implementation of the *k*-mismatch search is an optimized version of the algorithm depicted in Algorithm 2. Ψ-RA software can be downloaded from http://www.busillis.com/o_kulekci/PSIRA.zip for academic or noncommercial purposes.

## Results

The reads used in all experiments were collected from the SRR003078 experiment that is available from the sequence read archive at http://www.ncbi.nlm.nih.gov/sra. We mapped randomly selected 100K reads of various lengths from SRR003078 onto the complete human genome GRCh37 that is available at the site of Genome Reference Consortium (http://www.ncbi.nlm.nih.gov/projects/ genome/assembly/grc/human/index.shtml). All experiments were executed in a multiprocessor system of eight Intel Xeon 2.40 Ghz processors having a shared 32GB memory. The operating system was Gentoo running the Linux kernel 2.6.24.

We created the sparse suffix array-based indexes of the reference genome with sparsification factors of 4, 8, 12, and 16 according to the proposed methodology. The index sizes of Ψ-RA(4), Ψ-RA(8), Ψ-RA(I2), and Ψ-RA(I6), where Ψ-RA(*D*) refers to Ψ-RA with a sparsification factor of *D*, are 3.4GB, 2.0GB, 1.6GB, and 1.3GB respectively. Note that these values all include the 700MB complete human genome in 2-bit format.

**Figure 3:**
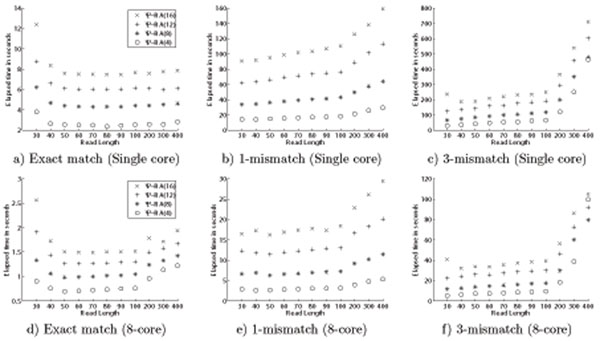
The effect of the sparsification factor D on single thread versus eight-thread executions.

SSAs give us the opportunity to tune the size of the index according to available memory by defining the sparsification factor *D.* A larger *D* value results in a smaller index size, but the computation cost increases as we need to consider offsets from 0 to *D* – 1. Figure 3 exhibits this trade off. The same queries were executed both with a single thread and with eight threads. It is observed that the gain in space is directly reflected in increased computation time, as expected. The increase in time can be compensated for with parallel execution of the software. Since today's processors in general have multiple cores, we can work with smaller-sized indexes and receive the same performance as if using a larger one. Sparse suffix arrays with larger sparsification factors match the performance of the smaller ones by benefiting from multicore architecture. For example on all cases, eight-core running of Ψ-RA(I6) is faster than single processor running of Ψ-RA(4) index.

**Figure 4:**
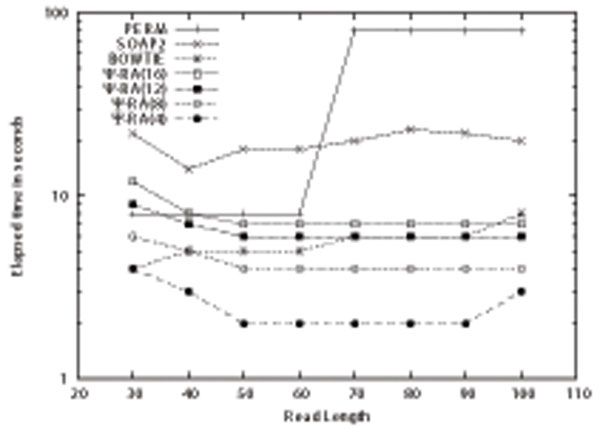
Exact matching comparison with some aligners.

Two widely used techniques in genome indexing are Burrows-Wheeler transform and seed-based hash tables. We compared Ψ-RA with Bowtie [5] and SOAP2 [21], as the two successful representatives of BWT genre, and with PerM [6] and mrsFast [8], which are based on spaced seeds and ordinary *q-*grams respectively. The index sizes of those aligners are 2.3GB (Bowtie), 6.1GB (SOAP2), 12.4GB (PerM), and 19.5GB (mrsFast). The exact matching performances of the tested aligners on mapping 100K reads against complete human genome are shown in Figure 4. The speed of the mrsFast was not competitive with the other aligners and hence it is not plotted.

**Figure 5:**
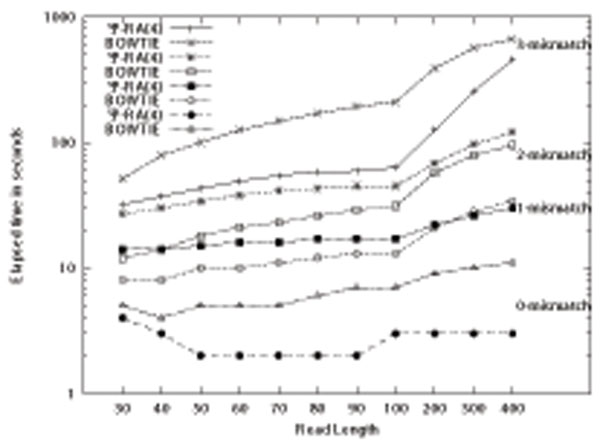
Performance comparison of Ψ-RA versus Bowtie on exact matching, 1-mismatch, 2-mismatch, and 3-mismatch alignments.

Short read aligners have different strategies and hence different parameters to perform matching. Although it is very difficult to make a fair benchmark, we compare the speed of Ψ-RA against Bowtie [5], which is one of the fastest aligners, to provide a comparison on the exact and also approximate matching performance. Figure 5 shows the results of the comparison. On exact matching and 3-mismatch, Ψ-RA(4) represents a better performance, where Bowtie is faster on 1- and 2-mismatch cases. We should note that although Bowtie does not guarantee the results for the 2- and 3-mismatch cases, Ψ-RA finds all occurrences.

## Conclusion

Indexing DNA sequences requires much memory. It is not possible to benefit much from the compressed indexes because of the random structure of the DNA sequences. As an alternative method of generating small size indexes of biological data, we focused on sparse suffix arrays and developed an aligner named Ψ-RA.

Ψ-RA is very fast on exact matching because of its cache-friendly structure. In addition to the speed caught in exact matching, we also integrated an elegant *k*-mismatch alignment capability by defining the rightmost mismatch criteria. The *k*-mismatch alignments of a queried read are reported according to the position of the mismatches, where being on the right is prioritized because the sequencing machines tend to generate erroneous bases particularly toward the end of the reads.

The size of a sparse suffix array changes with the chosen sparsification factor *D*, such that larger values result in smaller sized indexes. On the other hand, large sparsification factors worsen the time complexity of the processing. We showed experimentally that by applying parallelism in contemporary processor architectures, small-size indexes having large *D* match the performance of the large size indexes that have smaller *D* values. Ψ-RA gives users the flexibility to tune the size of the index according to the available resources. The index will fit in the main memory at the cost of increased computation time. That increase can be avoided up to some level in practice by benefiting from multicore processors.

## Authors contributions

Drs. M. Oğuzhan Külekci and Bojian Xu drafted the manuscript. The implementation and experiments were done by Dr. M. Oğuzhan Külekci. All authors contributed to the technical content of the study, including the algorithms for exact matching. Drs. Jeffrey Scott Vitter, M. Oğuzhan Külekci, and Bojian Xu worked on approximate matching. All authors read and approved the final manuscript.

## Competing interests

The authors declare no competing interests.
